# Primary intracranial extra-skeletal myxoid chondrosarcoma of right lateral ventricle with EWSR1 gene fusion: a case report and review of literature

**DOI:** 10.3332/ecancer.2021.1257

**Published:** 2021-06-30

**Authors:** Vinodh Kumar Selvaraj, Deleep Kumar Gudipudi, Rachna Khera, Sudha Murthy

**Affiliations:** 1 Department of Radiation Oncology, Basavatarakam Indo-American Cancer Hospital and Research Institute, Road number 10, Banjara Hills, Hyderabad-500034, Telangana, India; 2 Department of Pathology, Basavatarakam Indo-American Cancer Hospital and Research Institute, Road number 10, Banjara Hills, Hyderabad-500034, Telangana, India

**Keywords:** EWSR1 gene fusion, extra-skeletal myxoid chondrosarcoma, intracranial myxoid neoplasm, rare intracranial malignancy

## Abstract

**Background:**

Primary intracranial malignancies with extra-skeletal myxoid chondrosarcoma (EMC) features are extremely rare. EMC constitutes a distinct genomic entity characterised by reciprocal translocation of fusion genes, most commonly EWS RNA Binding Protein 1 (EWSR1) in 22q12 with Nuclear Receptor Subfamily 4 Group A Member 3 (NR4A3) in 9q2-q31.1. It is reported to have a high propensity for local recurrence and has potential for metastasis. So far in 28 years since its first description, only 17 cases of primary intracranial EMC were reported in literature. This would be the second case of intraventricular origin and first case from lateral ventricle.

**Case presentation:**

A 27-year-old male presenting with complaints of headache, seizures and pain in neck was diagnosed to have a mass lesion in right lateral ventricle in Magnetic Resonance Imaging of brain. He underwent right parieto-occipital craniotomy with total excision of the lesion. Initial histopathological examination was reported as Ependymoma, WHO grade II. However, blocks and slides review with immunohistochemistry (IHC) markers revealed neoplastic aetiology with extensive myxoid changes. Hence, fluorescent in-situ hybridisation (FISH) testing was done with EWSR1 break apart probe, which demonstrated EWSR1 break apart signals. Therefore, correlating the clinical findings with morphology, IHC and FISH, the diagnosis of primary intracranial EMC was rendered. Patient received adjuvant external beam radiation of 54 Gy in 30 fractions to the post-op region. At 29-month follow-up, there was no evidence of disease recurrence.

**Conclusions:**

Owing to the rarity of the condition, there are no standard treatment guidelines available for primary intracranial EMC. A combined treatment approach with surgery followed by adjuvant radiotherapy provides good local control with less morbidity.

## Background

Cartilaginous tumours constitute about 0.16% of intracranial neoplasms [1]. Primary cranial chondrosarcoma usually arises from synchondroses of skull base, especially the clival basioc ciput and the sphenoid bone [1–3]. Myxoid chondrosarcoma is a variant of chondrosarcoma which lacks hyaline cartilage areas and has cells arranged in cords in abundant myxoid stroma [4, 5]. They usually occur in lower extremities and very rarely present as primary intracranial lesion [6]. Over past 28 years, since the first description of myxoid chondrosarcoma in 1972, there are only 17 cases of intracranial extra-skeletal myxoid chondrosarcoma (EMC) reported in literature. The intracranial sites of origin were cerebellopontine angle, cerebellum, dura, fourth ventricle, pineal gland, falx cerebri and brain parenchyma [7–23]. Among the reported cases, there was only one case of intra-ventricular origin, which originated from fourth ventricle [7]. The case reported here will be the 18th case overall in literature, the second case of intra-ventricular origin and the first case of lateral ventricular origin. Here, we describe a case arising from right lateral ventricle in a 27-year-old male, initially thought to be ependymoma, treated with surgery followed by adjuvant radiotherapy. We also discuss when to consider EMC as a possible differential, pathological findings, differential diagnosis and treatment results.

## Case presentation

**Figure 1.**
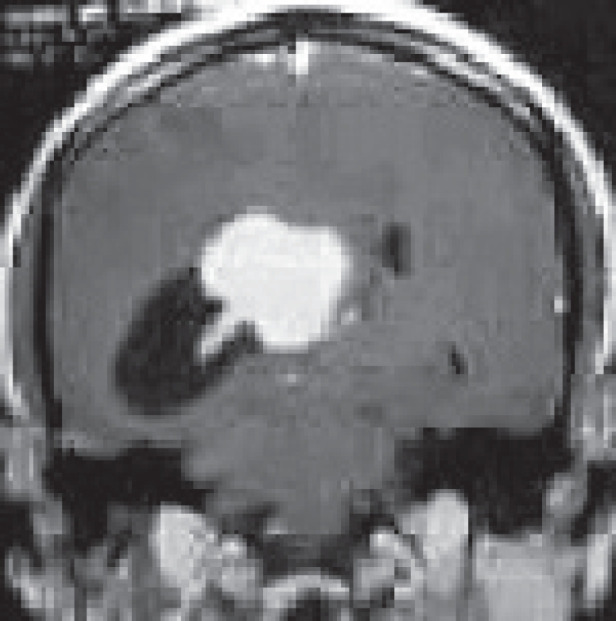
Pre-op MRI Brain, T2W coronal section showing a well-defined lobulated hyperintense mass lesion in atrium of the right lateral ventricle causing mass effect in form of dilated occipital horn of right lateral ventricle.

**Figure 2.**
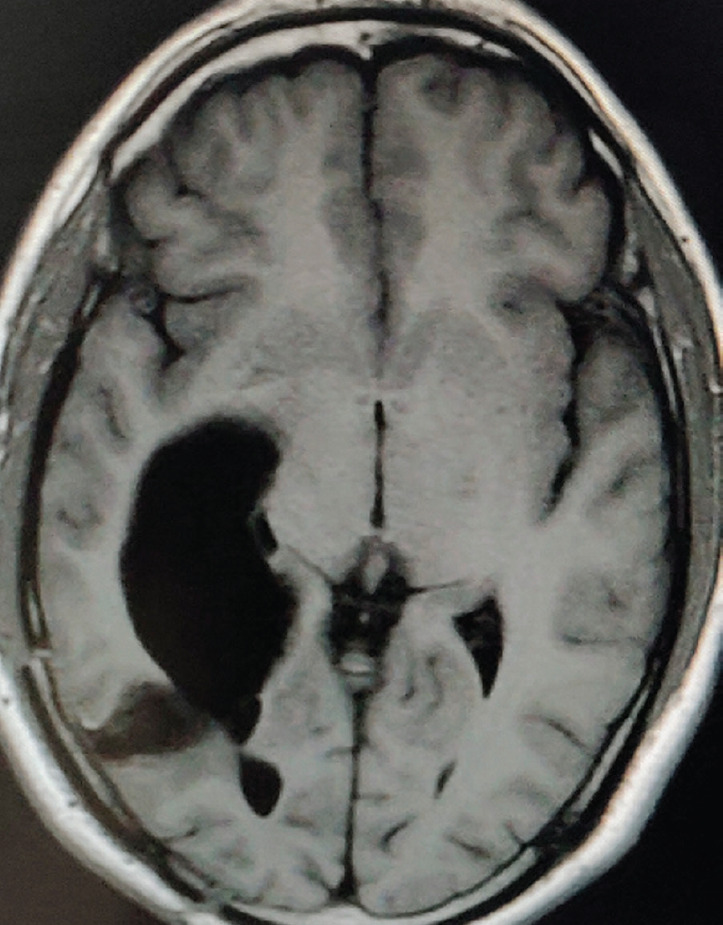
Post-op MRI Brain, T1W axial section showing a well-defined lobulated non-enhancing cystic lesion in right parietal lobe communicating with right lateral ventricle with dilated occipital horn.

A 27-year-old male evaluated elsewhere for complaints of headache, seizures and neck pain. Magnetic resonance imaging (MRI) of spine did not reveal any abnormality, whereas MRI brain showed a well-defined lobulated T1 hypointense, T2 heterogeneously hyperintense mass lesion of size 3.5×3.7×3.6 cm in atrium of the right lateral ventricle causing mass effect in form of dilated occipital and temporal horns of right lateral ventricle and midline shift of 11 mm towards left (Figure 1). Moderate perifocal oedema and homogenous intense enhancement on contrast administration were noted. Based on MRI findings, a differential diagnosis of choroid plexus papilloma, meningioma and sub-ependymal giant cell astrocytoma was considered. He underwent right parieto-occipital craniotomy with total excision of the lesion. Post-operative histopathological examination was suggestive of ependymoma, tanycytic variant, WHO grade II. He was then referred to our institute for adjuvant radiation. On clinical examination, he had no neurological deficit. MRI brain showed a well-defined lobulated non-enhancing cystic lesion of CSF intensity on all sequences in right parietal lobe communicating with right lateral ventricle with dilated temporal and occipital horn (Figure 2). There was no evidence of focal enhancing areas and was suggestive of post-operative gliosis. As the initial histopathological diagnosis was ependymoma, we performed a CSF cytology examination which was negative.

**Figure 3.**
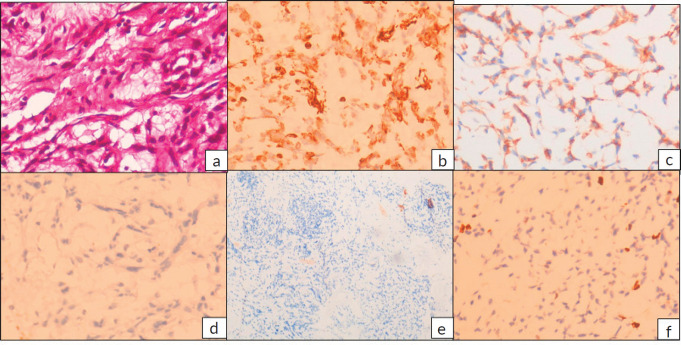
(a): Haematoxylin and eosin (H&E) stained sections showing tumour cells arranged in cords in abundant myxoid stroma. The tumour cells have ovoid nuclei and specked chromatin and moderate bipolar cytoplasm. (H&E 400×) IHC revealed tumour cells to be positive for (b): vimentin and (c): CD99, while (d): GFAP, (e): S100, CD34 and Pancytokeratin (not shown) were negative and (f): Ki-67 proliferation index was low, 1%–2%.

The postoperative blocks and slides were reviewed in our hospital. Histopathology revealed fragments of lesion with cells arranged in cords and focally in sheets in abundant myxoid stroma. The tumour cells were ovoid to spindle shaped with ovoid nuclei and specked chromatin and moderate bipolar cytoplasm. Focal perivascular arrangement of tumour cells was seen. No significant atypia, mitosis or necrosis identified. A provisional diagnosis of neoplastic aetiology with extensive myxoid change was rendered warranting immunohistochemistry (IHC) examination. IHC with vimentin, cluster of differentiation (CD99), revealed positive results, while Glial Fibrillary Acidic Protein (GFAP), pancytokeratin, CD34, S100 were negative and Ki67 proliferation index was low (1%–2%) (Figure 3). Subsequently fluorescent in-situ hybridisation (FISH) for EWS RNA Binding Protein 1 (EWSR1) gene rearrangement was carried out which revealed positive results. Final possibilities considered were myxoid chondrosarcoma or primary intracranial myxoid neoplasm with EWSR1 fusion.

Owing to the extremely rare occurrence of the disease, case was discussed in multi-disciplinary tumour board and planned for post-operative radiation. Patient was immobilised with a thermoplastic head mask in a supine position and contrast-enhanced CT was taken at 3 mm thickness on a Philips Big bore CT Scan machine. Images were exported to ECLIPSE Treatment Planning system. Pre-op and post-op MRIs were fused with the Planning CT images. Contouring was done on fused images, post-operative bed along with organ at risk (eyes, brainstem, temporal lobe, optic chiasm, lens, cochlea, optic nerves and pituitary gland) was delineated. A planning target volume was generated around the clinical target volume. A dose of 54 Gy in 30 fractions at 1.8 Gy per fraction using 6 MV photons was delivered to the patient using Rapid-Arc image-guided radiotherapy technique. Patient tolerated treatment well with grade 1 skin reactions and alopecia at the treated site. At 29 months follow-up, patient had no neurological deficit and MRI brain showed no evidence of disease recurrence.

## Discussion

Extra-skeletal cartilaginous tumours are thought to be derived from primitive mesenchymal cells or embryonal rests of cartilaginous matrix within the bone or a result of metaplasia of fibroblasts [24, 25]. Histologically, there are three variants extra-skeletal cartilaginous tumours: classical, mesenchymal and myxoid [4, 5]. Classical variant is rare, tends to occur in older age group and has a better prognosis [25–27]. Whereas mesenchymal variant usually occurs in younger age group and has higher propensity for frequent local recurrences or metastatic disease [27–29]. Myxoid chondrosarcoma was first described by Enziner and Shiraki in 1972 as a deep soft tissue tumour of the extremities [30]. EMC is a distinct genomic entity with an indolent course but with potential for local recurrence and distant metastasis [31]. Sometimes, it is found to be associated with hereditary syndromes such as Ollier disease and Maffucci syndrome [27, 32].

The symptomatology of EMC is extremely non-specific. It is consistent with any brain lesion with headache, seizures, focal neurological deficit and symptoms due to mass effect/midline shift. MRI is the standard imaging modality of choice. In MRI, primary intracranial EMCs are seen as hypointense well-demarcated lesions in T1-weighted images with slightly higher signal intensity in T2-weighted images and generally lack surrounding oedema [10, 12–14]. The close differentials to be considered are meningioma or metastatic disease [18, 33]. Therefore, it is always mandatory to make a histological diagnosis before treatment. IHC panel of markers such as vimentin, cytokeratin, S100 and GFAP play a vital role in histopathological diagnosis. Vimentin positivity and cytokeratin negativity were reported in most cases of primary intracranial EMC in literature [14, 17, 18, 21]. S100 negativity helps to rule out chordoma [34]. EWSR1 gene fusion in FISH testing with IHC markers favours the diagnosis of EMC.

In the case discussed above, the patient presented with headache, seizures and neck pain. Both clinical and MRI findings were non-specific. A diagnosis of EMC was never considered at any stage of clinical evaluation. The morphologic diagnosis of ependymoma was rendered at a community centre presumably based on ovoid cells with few bland elongated nuclei and location of the tumour being lateral ventricle. However, when it was reviewed at our centre, the young age, bland appearance of tumour cells which were round to ovoid in a myxoid matrix associated with cord like arrangement, negativity for GFAP & epithelial markers, and strong vimentin & CD99 (membrane) positivity by IHC led us to consider this to be a sarcoma, particularly a myxoid sarcoma (myxoid liposarcoma, myxofibrosarcoma, myxoid angiomatoid fibrous histiocytoma (AFH), intracranial myxoid sarcoma with EWSR1 translocations). Since we had the EWSR1 break apart probe, we were compelled to do FISH for EWSR1 which demonstrated EWSR1 break apart signals. The differential diagnosis at this juncture consisted of all intracranial myxoid lesions with EWSR1 translocation. The possibility of myxoid (AFH) was ruled out as there was no lymphocytic cuffing or prominence of vasculature. Myxoid liposarcoma was also ruled out due to morphology of cells that lack lipoblasts and no S100 staining. The possibility of myoepithelial carcinoma needs to be ruled out in this setting which was negated by cytokeratin AE1/AE3 and S100 expression by IHC. The other fusion partners of EWSR1 in intracranial myxoid sarcomas include cyclic-AMP response element-binding protein 1 (CREB1), cyclic-AMP response element modulating protein (CREM) and Activating Transcription Factor 1 (ATF1) of which CREB1 being the most common. The Proliferation index (Ki 67) also was substantially low which corroborated with the indolent behaviour of EMC, presumably a tumour with EWSR1-Nuclear Receptor Subfamily 4 Group A Member 3 (NR4A3) fusion. However, NR4A3 by FISH was not available at any centre in the country at that point in time. Therefore, correlating the clinical findings with morphology, IHC and FISH, the diagnosis of EMC was rendered.

About two thirds of EMC show chromosomal reciprocal translocation t (9;22) q22: q12 resulting in the fusion of EWSR1 gene to NR4A3 [35–37]. A smaller proportion of EMC tumours are known to harbour TATA-Box Binding Protein Associated Factor 15 (TAF15)-NR4A3 fusions which are known to show high grade features morphologically. The other fusion partners of EWSR1 in intracranial myxoid sarcomas include CREB1, CREM and ATF1 with CREB1 being the most common. Custom made probes for NR4A3 gene were used for NR4A3 locus on chromosome 9q22 in a case report of EMC to study EWSR1-NR4A3 fusions [37].

On literature review, surgery has been the first treatment modality of choice for primary intracranial EMC. Either total or subtotal resection of tumour was done in all cases depending on site and extent of the tumour (Table 1) [7–23]. Adjuvant radiotherapy was advocated in most of the cases. Different modalities of radiotherapy such as radioactive iodine brachytherapy, external beam photon therapy or proton therapy were utilised (Table 1). As summarised in Table 1, the adjuvant radiation dose with photons ranged from 54–60.8 Gy in 28–33 fractions [10, 14–16, 18, 21, 22]. In a single case treated with protons, a dose of 66 CGE was delivered [20]. Adjuvant chemotherapy such as ifosfamide and temozolomide was administered in two patients but their role in EMC is doubtful [18, 21]. Therefore, in the case reported, we delivered adjuvant radiotherapy of 54 Gy in 30 fractions following total surgical resection of the lesion done elsewhere.

## Conclusions

Primary intracranial EMC is an extremely rare malignancy of central nervous system. It is always advisable to do a thorough histopathological examination with IHC markers in all intracranial neoplasms to identify the true histology. EMC should be considered as one of the differential diagnosis, especially when tumour cells appear bland in myxoid matrix with associated cord like arrangement and suggestive IHC markers. Combined modality of treatment with surgery followed by adjuvant radiotherapy results in good local control.

## Ethical approval and consent to participate

Not applicable.

## Consent for publication

Consent for publication was obtained from the patient.

## Competing interests

The authors declare that they have no competing interests.

## Funding

The authors declare that no specific grant was obtained for this research from any funding agency in the public, commercial or not-for-profit sectors in the design of the study and collection, analysis and interpretation of data and in writing the manuscript.

## Authors’ contributions

Dr Deleep Kumar Gudipudi contributed to concept, design and proof reading of manuscript. Dr Vinodh Kumar Selvaraj contributed to data collection, review of literature and manuscript writing. Dr Rachna Khera and Dr Sudha Murthy contributed to preparing the histopathology images and in manuscript writing of the pathologic description of the case.

## Figures and Tables

**Table 1.** Primary intracranial EMC cases reported in literature.

First author/year	Age (year)/sex	Location	Surgery	Adjuvant treatment	Outcome
Scott *et al* [7]	39/M	Fourth ventricle	STR	Nil	13 days, died (ventriculitis)
Smith and Davidson [8]	12/M	Cerebellum and meninges	CR	Nil	13 months, no recurrence
Salcman *et al* [9]	28/F	Left parafalcine and dura of falx	CR	Nil	At 10 months, recurrence – Surgery followed by radioactive 125I Brachytherapy 20 months, no recurrence, alive
Sato *et al* [10]	43/F	Pineal gland and dura	STR	RT (60 Gy in 30 fractions) and chemotherapy	3 years, tumour progression, died
Sala *et al* [11]	55/F	Petrooccipital dura	CR	Nil	Recurrence at 10, 16, 31 and 43 months – Repeated surgery 7 years, died
Gonzalez- Lois *et al* [12]	17/F	Frontotemporal lobe and dura	CR	Nil	At 16 months recurrence – Surgery followed by RT (60 Gy in 30 fractions). 20 months, no recurrence, alive
Chaskis *et al* [13]	69/M	Left parietal lobe	CR	Nil	1 month, died (septic shock)
Im *et al* [14]	43/M	Left parietal lobe	CR	RT (59.4 Gy in 33 fractions)	3 years, no recurrence, alive
Sorimachi *et al* [15]	37/F	Pineal gland	STR	Nil	At 13 months, recurrence – Surgery (CR). 7 months, no recurrence, alive
O’Brien *et al* [16]	26/F	Left cerebellopontine angle	STR	RT (Proton therapy)	1 year, no tumour progression, alive
Park *et al* [17]	21/F	Right thalamus	CR	RT (60.8 Gy)	6 months, no recurrence, alive
Dulou *et al* [18]	70/F	Left frontal lobe	CR	RT (60 Gy in 30 fractions) + Chemotherapy (ifosfamide)	10 months, died
Govind *et al* [19]	40/M	Left frontal lobe	CR	Nil	6 months, no recurrence, alive
Rodgers *et al* [20]	16/M	Right thalamus	STR	Re-resection followed by proton therapy (30.67 CGE to primary site and 36.30 CGE to cranio-spinal axis) & Pazopanib	16 months, died
Qin et al [21]	41/F	Left cerebellum	STR	RT (56 Gy in 28 fractions) + Chemotherapy (Temozolomide)	19 months, no recurrence, alive
Rashed *et al* [22]	65/F	Left parietooccipital space	STR	RT (60 Gy in 30 fractions)	6 months, died
Akakin *et al* [23]	35/F	Falx cerebri	CR	NR	Recurrence at 2 months – Surgery followed by RT (18 Gy in 1 fraction). At 9 months from previous treatment – second recurrence: Surgery followed by RT (15 Gy in 1 fraction)
Present case	27/M	Right lateral ventricle	CR	Adjuvant RT (54 Gy in 30 fractions)	At 29 months, no recurrence, alive

F, Female; M, Male; CR, Complete resection; STR, Sub-total resection; RT, Radiotherapy; Gy, Gray; CGE, Cobalt gray equivalent
